# Models of Neocortical Layer 5b Pyramidal Cells Capturing a Wide Range of Dendritic and Perisomatic Active Properties

**DOI:** 10.1371/journal.pcbi.1002107

**Published:** 2011-07-28

**Authors:** Etay Hay, Sean Hill, Felix Schürmann, Henry Markram, Idan Segev

**Affiliations:** 1 Interdisciplinary Center for Neural Computation and Edmond and Lily Safra Center for Brain Sciences, The Hebrew University, Jerusalem, Israel; 2 Brain Mind Institute, Ecole Polytechnique Fèdèrale de Lausanne (EPFL), Lausanne, Switzerland; 3 Department of Neurobiology, The Hebrew University, Jerusalem, Israel; Université Paris Descartes, Centre National de la Recherche Scientifique, France

## Abstract

The thick-tufted layer 5b pyramidal cell extends its dendritic tree to all six layers of the mammalian neocortex and serves as a major building block for the cortical column. L5b pyramidal cells have been the subject of extensive experimental and modeling studies, yet conductance-based models of these cells that faithfully reproduce both their perisomatic Na^+^-spiking behavior as well as key dendritic active properties, including Ca^2+^ spikes and back-propagating action potentials, are still lacking. Based on a large body of experimental recordings from both the soma and dendrites of L5b pyramidal cells in adult rats, we characterized key features of the somatic and dendritic firing and quantified their statistics. We used these features to constrain the density of a set of ion channels over the soma and dendritic surface via multi-objective optimization with an evolutionary algorithm, thus generating a set of detailed conductance-based models that faithfully replicate the back-propagating action potential activated Ca^2+^ spike firing and the perisomatic firing response to current steps, as well as the experimental variability of the properties. Furthermore, we show a useful way to analyze model parameters with our sets of models, which enabled us to identify some of the mechanisms responsible for the dynamic properties of L5b pyramidal cells as well as mechanisms that are sensitive to morphological changes. This automated framework can be used to develop a database of faithful models for other neuron types. The models we present provide several experimentally-testable predictions and can serve as a powerful tool for theoretical investigations of the contribution of single-cell dynamics to network activity and its computational capabilities.

## Author Summary

The pyramidal cell of layer 5b in the mammalian neocortex extends its dendritic tree to all six layers of cortex, thus receiving inputs from the entire cortical column and supplying the major output of the column to other brain areas. L5b pyramidal cells have been the subject of extensive experimental and modeling studies, yet realistic models of these cells that faithfully reproduce both their perisomatic Na^+^ and dendritic Ca^2+^ firing behaviors are still lacking. Using an automated algorithm and a large body of experimental data, we generated a set of models that faithfully replicate a range of active dendritic and perisomatic properties of L5b pyramidal cells, as well as the experimental variability of the properties. Furthermore, we show a useful way to analyze model parameters with our sets of models, which enabled us to identify some of the mechanisms responsible for the dynamic properties of L5b pyramidal cells as well as mechanisms that are sensitive to morphological changes. This framework can be used to develop a database of faithful models for other neuron types. The models we present can serve as a powerful tool for theoretical investigations of the contribution of single-cell dynamics to network activity and its computational capabilities.

## Introduction

Neocortical pyramidal cells in layer 5 (L5 PCs) are important input-output units in the cortical column. Their dendrites span the entire column, thus receiving input from all six layers, and the cells provide the major output from the column to various parts of the brain. These cells are divided into two main classes that differ in dendritic morphology, electrical properties, axonal projections that they typically make [1]–[2], thalamocortical input they receive [3], and location of their soma within layer 5. L5b PCs are pyramidal cells of the deeper part of the layer (L5b). These thick-tufted cells project to subcortical targets such as the tectum, brainstem and spinal cord, and they tend to discharge a short burst of spikes in the beginning of a spike train. By contrast, the thin-tufted pyramidal cells of the superficial part of the layer (L5a) discharge spikes with no adaptation, and project to other parts of the cortex [4].

Due to the large diameter of their apical dendrites, L5b PCs are readily available for intracellular dendritic recordings and as such they have been extensively studied over the past few decades. Previous works characterized numerous active properties of the cells apical dendrites [5]–[8] and recently also the basal dendrites [9]–[11], as well as the ionic currents involved and partially also the spatial distribution of the underlying ion channels over the dendritic surface [12]–[13]. Such active dendritic properties are suggested to play a key role in information processing [14], non-linear computations [15]–[16] and synaptic integration [17]–[18]. Recent experiments have also highlighted the impact of L5 PCs on sensation and action in anaesthetized [19] and in behaving [20] animals.

The key active properties of L5b PCs involve two main spiking zones. Na^+^ action potentials (APs) are initiated at the perisomatic region with a typical frequency-current (f–I) relation and firing response to a prolonged suprathreshold step current (perisomatic step current firing) [4]. The second spiking zone is located at the distal apical dendrites [7], [21]–[22], where Ca^2+^ spikes are generated in response to an intense dendritic [22] or somatic [23] stimulation *in vitro*. Recent *in vivo* studies demonstrate a correlation between dendritic Ca^2+^ signals and sensation [24] or wakefulness [25]. Importantly, *in vitro* studies have demonstrated that the two spiking zones interact with each other, whereby the coincidence of the back-propagating action potential (BAP) and local excitatory postsynaptic potential (EPSP) at the distal dendrites triggers a dendritic Ca^2+^ spike, which in turn triggers one or more additional perisomatic Na^+^ APs [6]. This BAP-activated Ca^2+^ spike (BAC) firing highlights the interplay between the two spiking zones, and may be involved in coincidence detection of EPSPs and APs [26], and in spike timing-dependent plasticity [27]–[28].

While there are various conductance-based models of L5b PCs, they are either hand-tailored to capture only a few particular properties of these cells, such as the back propagation of action potentials [29] or the effect of I_h_ on input integration [13], or they are meant to explore a general idea rather than faithfully replicate specific dynamical properties [30]. A notable existing model was developed by Schaefer et al. [31] and is capable of replicating the BAC firing; however it does not produce the typical f–I curve and perisomatic step current firing behavior of these cells. Kole et al. [32] developed a model that fits the shape of a single spike at the axon, soma and dendrites, but is incapable of generating apical Ca^2+^ spikes and does not capture the f–I curve or perisomatic step current firing. Another significant modeling effort did manage to reproduce some features of the response to suprathreshold current steps recorded at the soma and apical dendrites [33], or only features of the Na^+^ excitability in the apical dendrites [34]. However, these models do not attempt to reproduce dendritic Ca^2+^ spikes or BAC firing, and the relevant channels are missing from the apical dendrites. Presently, there is still no model for L5b PCs that faithfully replicates both of the two basic firing behaviors, one at the soma and the other at the apical dendrite (but see an important effort in this direction for hippocampal CA1 PCs [35]).

In this work we extended our theoretical framework [36] of utilizing an automated fitting method, the multi-objective optimization (MOO) algorithm combined with an evolutionary algorithm, to constrain the features of the firing and shape of spikes (for a review on automated fitting of models to experiments, see [37]–[38]). We targeted local spikes both in the soma and distal apical tree, as well as the interaction between the two zones. We arrived at models that faithfully capture the main active properties of mature L5b PCs, including their experimental variability, as quantified by feature statistics over this cell type. We also demonstrate that an inspection of parameter value ranges in our sets of models provides useful insights into the key ionic conductances that underlie the active properties of L5b PCs, as well as mechanisms that are sensitive to morphological changes. We propose that our MOO framework can be used to extend these models for both L5b PCs as well as other neuron types when further experimental data becomes available. Eventually, this approach will provide a database of neuron models that capture the key properties of all neuron types, including their experimental variability. Our realistic models can serve as a powerful tool for theoretical investigations of the contribution of single-cell dynamics to the overall network dynamics and its emergent computational capabilities.

## Results

### Features of perisomatic and dendritic firing in L5b PCs

We defined the features of the perisomatic and dendritic firing behaviors that we intended our models to replicate (the “target” firing behaviors) and quantified their mean and standard deviation (SD, see Table 1) using experimental voltage traces recorded in several mature L5b PCs or data reported in the experimental literature (see Methods). For the target of perisomatic step current firing, we used ten somatic features of the average response to three normalized (see Methods) current step amplitudes (Table 1, leftmost four columns). Evidently, some perisomatic firing features such as the AP half-width, AP peak and inter-spike interval (ISI) adaptation did not differ significantly over different step amplitudes, in contrast to features such as the first spike latency and ISI-CV for the spike train. With increasing current, the spike train became more regular (as quantified by the ISI-CV feature), the latency of the first spike decreased and the initial burst's ISI became less variable (see also [4]). For the target of BAC firing we used ten dendritic and somatic firing features (Table 1, rightmost two columns). The experimental traces indicated a rather robust Ca^2+^ spike height and width during BAC firing, and a more variable ISI for the resulting burst of perisomatic Na^+^ APs. The experimentally observed amplitude and variability of the BAP at two distal apical locations also served to constrain the model BAC firing.

**Table 1:** Mean and SD values of features of perisomatic step current firing and of BAC firing.

Features of perisomatic step current firing				Features of BAC firing	
Feature	Mean±SD, Low frequency	Mean±SD, Reference frequency (15 Hz)	Mean±SD, High frequency	Feature	Mean±SD
1. Spike frequency (Hz)	9±0.88	14.5±0.56	22.5±2.22	1. Ca^2+^ spike peak (mV)	6.73±2.54
2. Adaptation Index	0.0036±0.0091	0.0023±0.0056	0.0046±0.0026	2. Ca^2+^ spike width (ms)	37.43±1.27
3. ISI-CV	0.1204±0.0321	0.1083±0.0368	0.0954±0.0140	3. Somatic AP spike count (during somatic + dendrite current injection)	3±0
4. Initial Burst ISI (ms)	57.75±33.48	6.625±8.65	5.38±0.83	4. Mean somatic AP ISI (ms)	9.9±0.85
5. First spike latency (ms)	43.25±7.32	19.13±7.31	7.25±1	5. Somatic AHP depth (mV)	−65±4
6. AP peak (mV)	26.23±4.97	16.52±6.11	16.44±6.93	6. Somatic AP peak (mV)	25±5
7. Fast AHP depth (mV)	−51.95±5.82	−54.19±5.57	−56.56±3.58	7. Somatic AP half-width (ms)	2±0.5
8. Slow AHP depth (mV)	−58.04±4.58	−60.51±4.67	−59.99±3.92	8. Somatic AP spike count (during somatic current injection only)	1±0
9. Slow AHP time	0.238±0.030	0.279±0.027	0.213±0.037	9. BAP amplitude at 620 µm (mV)	45±10
10. AP half-width (ms)	1.31±0.17	1.38±0.28	1.86±0.41	10. BAP amplitude at 800 µm (mV)	36±9.33

Leftmost four columns–features of perisomatic step current firing, for three different step amplitudes yielding low, medium and high firing rates. Rightmost two columns–features of BAC firing. Statistics reflect several cells. In constraining models for BAC firing, we used features of the dendritic Ca^2+^ spike and somatic Na^+^ APs in response to coincident somatic and dendritic current injections, as well as BAP attenuation during current injection only to the soma (See Methods for details).

### Models constrained by either BAC firing or perisomatic step current firing

We first fitted, separately, either the BAC firing target or the perisomatic step current firing target and explored their respective conductance mechanisms. We selected key conductance mechanisms found in L5b PCs or generally in neocortical neurons [39] and well-characterized experimentally (see Methods). In optimizing either target, we used the same set of 22 free parameters (Table 2), primarily the densities of the conductance mechanisms. The density of I_h_ conductance was not a free parameter but rather distributed similarly in all optimizations based on previous studies (see Methods). This distribution of I_h_ ensured that all our models exhibited key subthreshold properties of L5b PCs such as I_h_ related effects on EPSP summation [40]–[43], as well as a resting membrane potential gradient along the apical tree, with a slope of 10 mV/mm [7], [13].

**Table 2:** Parameter limits for ion channel density and Ca^2+^ dynamics parameters used in the evolutionary algorithm.

Parameter	Lower Limit	Upper Limit	Parameter	Lower Limit	Upper Limit
s. 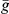 _ Nat_	0	40,000	ax. 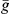 _leak_	0.2	0.5
s. 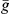 *_Nap_*	0	100	b. 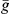 _leak_	0.15	0.5
s. 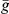 *_Kp_*	0	10,000	a. 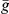 _leak_	0.15	0.5
s. 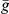 *_Kt_*	0	1,000	a. 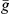 _Nat_	0	200
s. 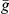 *_Kv3.1_*	0	20,000	a. 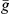 *_Kv3.1_*	0	200
s. 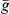 *_Ca_HVA_*	0	10	a. 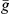 *_Ca_HVA_*	0	25
s. 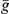 *_Ca_LVA_*	0	100	a. 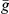 *_Ca_LVA_*	0	1000
s. 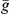 *_SK_*	0	1,000	a. 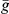 *_SK_*	0	50
s.τ_decay_	20	1,000	a. 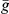 _m_	0	5
s. γ	0.0005	0.05	a.τ_decay_	20	200
s. 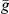 _leak_	0.2	0.5	a. γ	0.0005	0.05

s–soma, a–apical, b–basal, ax–axon. Conductance is in pS/µm^2^, τ_decay_ is in ms. Values of apical Ca^2+^ channels are given for the high density distal zone.

**Figure 1:**
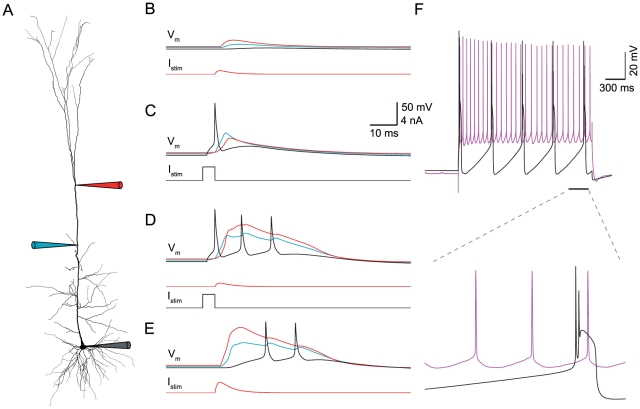
Models constrained only by BAC firing may fail to respond properly to perisomatic step current. **A.** Reconstructed morphology of an L5b neocortical pyramidal cell, age p36, used for the fitting and simulations. Recording and stimulation sites are indicated by schematic electrodes at the soma (black), proximal apical dendrite (400 µm from the soma, blue) and distal apical site (620 µm from the soma, red). **(B–E).** Model simulation of BAC firing (compare to [6]). **B**. EPSP-like current injection at the distal dendrites (I_stim_, red trace) with peak amplitude of 0.5 nA produced a subthreshold depolarization of 2.5 mV at the soma (V_m_, black trace). **C.** Suprathreshold step current at the soma evoked a somatic AP that back-propagated into the dendrites. **D.** BAC firing. The combination of somatic and dendritic current injection (separated by an interval of 5 ms) evoked a dendritic Ca^2+^ spike (V_m_, red trace) followed by a burst of two additional somatic APs. **E.** Dendritic Ca^2+^ spike could be evoked by an intense (2.5 nA) current injection to the distal dendrite alone, also evoking two APs at the soma. **F.** Top: the model's firing response to somatic step current injection did not agree with the experimentally observed response (black–model, magenta–experiment). Bottom: 300 ms of the response corresponding to the bar in the top part.

We first constrained models only by features of the BAC firing (Table 1, rightmost two columns), generating a set of 899 acceptable models (see definition in Methods). Figure 1 depicts the firing behavior of one example model from the set. An EPSP-like current with 0.5 ms rise time, 5 ms decay time and amplitude of 0.5 nA injected at the model distal apical dendrites (620 µm away from the soma) resulted in a local EPSP of 14 mV and a somatic EPSP of 2.5 mV (Figure 1B). A brief 5 ms, 1.9 nA suprathreshold current injected in the model soma yielded an AP that back-propagated to the dendrites (Figure 1C), decaying to yield the BAP amplitude at the main bifurcation which is within the experimental range [44]. When both somatic and dendritic stimuli coincided within 5 ms, the model neuron generated BAC firing with a large and broad Ca^2+^ spike followed by a burst of two additional somatic Na^+^ APs (Figure 1D). Intense stimulation of the distal dendrites alone (Figure 1E) was sufficient to produce a dendritic Ca^2+^ spike and two somatic Na^+^ APs. These behaviors are in full agreement with the experimental literature [6]. The model Ca^2+^ spike peak and width were within 0.86 SD from the experimental mean; the BAP amplitudes were within 1.4 SD from the experimental mean; and the perisomatic AP ISI was within 1.1 SD from the experimental mean. Models in the set had feature values ranging within 2–3 SD (our designated cut-off for acceptable models) from the experimental mean, thus exhibiting the experimental variability.

Importantly, acceptable models that were constrained to only replicate the BAC firing were not guaranteed to faithfully replicate the firing response to prolonged somatic depolarizing current step (Figure 1F). In the example model described above, the frequency of Na^+^ APs at the soma was too low (Figure 1F, top) and the shape of individual spikes within the train did not resemble that of the experimental spikes (Figure 1F, bottom). Hence, fitting the BAC firing target behavior did not ensure good performance on our other target behavior (see also [45]).

**Figure 2:**
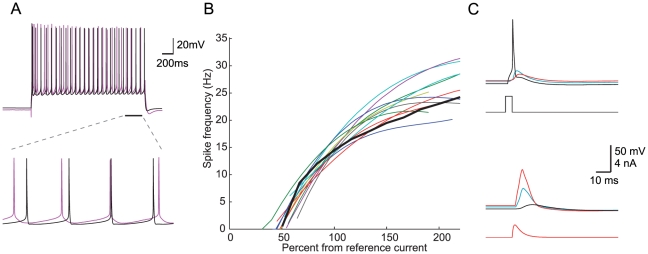
Models constrained only by perisomatic step current firing are not guaranteed to generate BAC firing. **A.** Top: model (black) response to a 2 second 793 pA depolarizing step current at the soma as compared to the corresponding experimental trace (magenta). Bottom: 300 ms of the response corresponding to the bar in the top part. **B.** f–I curve for the 11 L5b PCs (colors) and for the model (black). **C.** The apical tree in this model was only weakly excitable, exhibiting no active BAP (top), and was incapable of generating a dendritic Ca^2+^ spike even under intense distal stimulation (bottom). All measures and definitions in C are as in Figure 1.

Next, we constrained models only by features of the perisomatic step current firing (Table 1, leftmost four columns), and arrived at a second set of 52 acceptable models. For an example model from the set, a qualitative comparison of the experimental and simulated model response to depolarizing current step is shown in Figure 2A, demonstrating the similarity in spike train features (frequency, latency, initial burst, ISI-CV and adaptation index) and spike shape features (spike height, after-hyperpolarization, and spike width). The values of all features in that model were within 1–2 SD from the experimental mean, except for the first spike latency that was within 3 SD from the experimental mean. In addition, the whole f–I curve of the model fell within the range of the experimental f–I curves (Figure 2B), demonstrating that matching model parameters to only three selected points in the f–I curve was sufficient to constrain the entire f–I curve. Models in the set had feature values ranging within 2–3 SD from the experimental mean, thus exhibiting the experimental variability. As expected, these models were not guaranteed to capture the active dendritic properties, despite having the same free parameters on the apical dendrites as those used for fitting BAC firing (Figure 1). In the example model shown in Figure 2, dendrites were only weakly excitable, resulting in a strongly attenuated, essentially passive, BAP (Figure 2C, top), and failure to produce a Ca^2+^ spike even under intense distal apical stimulation (Figure 2C, bottom).

**Figure 3:**
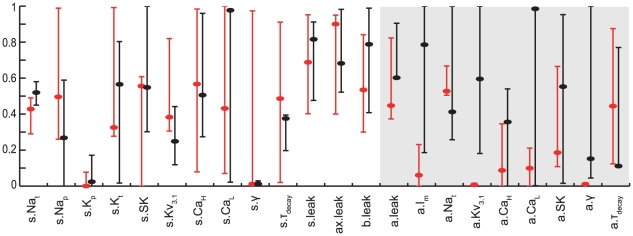
Parameter ranges for acceptable models for either perisomatic step current firing or BAC firing. Distribution of normalized parameter values in models constrained by BAC firing (red, n = 899 acceptable models) or by perisomatic step current firing (black, n = 52 acceptable models). For ease of viewing, the graph region containing parameters at the apical tree is shaded in gray. Red and Black circles correspond to specific normalized parameter values of the models shown in Figures 1 and 2, respectively. Real values for the different ion conductances can be derived by referring to the upper limits given in Table 2.

To highlight mechanisms that provide acceptable models for BAC firing or for perisomatic step current firing, we compared the range of each model parameter in the two corresponding sets of models (Figure 3). Evidently, the ranges of most apical parameters (shaded area in Figure 3) were markedly smaller when constraining for BAC firing alone (Figure 3, red lines) than when constraining only for perisomatic step current firing (Figure 3, black lines). Thus, faithful models for BAC firing required the density of Ca^2+^, Na^+^ and K^+^ ion channels in the apical tree, as well as the parameters of Ca^2+^ dynamics, γ and τ_decay_ (see Methods), to be within a rather tight range (Figure 3, red lines in shaded area). For example, the range for the density of apical Na_t_ was 101–133.5 pS/µm^2^ when constraining for BAC firing (Figure 3, red lines), and 51.5–200 pS/µm^2^ (almost spanning the entire free parameter range) when not constraining for BAC firing (Figure 3, black lines). The ranges of I_m_, Kv_3.1_, Ca_HVA_, Ca_LVA_, SK, γ and τ_decay_ in models for BAC firing were 0–1.155, 0–3.05, 0–8.65, 0–211, 25–29.5 pS/µm^2^, 5e-4–5.85e-4 and 24.6–175 ms, respectively. Conversely, in models for perisomatic step current firing the ranges of somatic parameters such as Ca_HVA_ density, Ca^2+^ dynamics γ and τ_decay_ were smaller, and the ranges of SK and Na_p_ densities were shifted, compared to models for BAC firing. Other somatic parameter ranges in both sets of models were similar (see Discussion). We note that the range of acceptable density of apical Na_t_ given above agrees with previous experiments [12], and that the densities of the other dendritic channels in acceptable BAC firing models are in agreement with previous theoretical estimates [30]–[31].

In order to understand why the models shown in Figures 1 and 2 failed in the targets they were not constrained with, we related the particular parameter values of a model for one target to the ranges delineated by the set of acceptable models for the other target. The red and black circles in Figure 3 correspond to the normalized parameter values of the models shown in Figures 1 and 2, respectively.

First, we looked for dendritic parameter values of the model for perisomatic step current firing (Figure 3, black circles) that were outside the parameter ranges delineated by the set of acceptable models for BAC firing (Figure 3, red ranges), and therefore might underlie the failure of the model to generate a Ca^2+^ spike or properly back-propagate APs (Figure 2C). For example, the model's apical Na_t_ density (82.5 pS/µm^2^) was below the acceptable range for BAC firing (Figure 3, red range, corresponding to 101–133.5 pS/µm^2^), and its apical Kv_3.1_ density (119 pS/µm^2^) was above the acceptable range (0–3.05 pS/µm^2^). Either of the deviant densities was therefore likely to be the reason for the model's failure to properly back-propagate APs to the apical tree [12]. We verified this hypothesis either by reducing the apical Na_t_ density in the acceptable model for BAC firing (Figure 3, red circles, corresponding to 105.5 pS/µm^2^) to this low value, which resulted in a similar failure to back-propagate APs; or by increasing the value of this channel density in the model for perisomatic step current firing to acceptable values (Figure 3, red range), which resulted in a more acceptable BAP (not shown). Several parameters may underlie the failure of the model to generate a Ca^2+^ spike. Its apical I_m_ and Kv_3.1_ densities (3.9 and 119 pS/µm^2^, respectively) as well as the apical γ value (7.59e-3) were above the acceptable ranges for BAC firing (0–1.155, 0–3.05 pS/µm^2^, and 5.00e-4-5.85e-4, respectively). We tested this hypothesis by shifting the values of these three parameters in the model to those of the acceptable model for BAC firing, and indeed observed Ca^2+^ spike under distal apical stimulation (not shown) as in models for BAC firing (Figure 1E).

A similar examination of the parameter values of the model for BAC firing (Figure 3, red circles) showed that the model had apical I_m_ and Kv_3.1_ densities (0.3 and 1.49 pS/µm^2^, respectively) below the acceptable ranges for perisomatic current step firing (Figure 3, black ranges, corresponding to 0.93–5 and 36.2–200 pS/µm^2^, respectively), and somatic Ca^2+^ dynamics τ_decay_ (486 ms) above the acceptable range (197–395 ms). These aberrant values may have increased the somatic and dendritic excitability as suggested by the bursts seen in the response to somatic step current (Figure 1F). We tested the model when shifting the apical Kv_3.1_ and I_m_ densities and somatic τ_decay_ to the values of the acceptable model for perisomatic step current firing (Figure 3, black circles) and managed to significantly improve the response (not shown).

We found further evidence that active dendritic mechanisms did not play a significant role in perisomatic step current firing, by comparing somatic parameters in our models for this target behavior to models that optimized the same target but without the free apical parameters. The dendrites in the latter models were therefore essentially passive, having only I_h_ and leak conductance. We found that the somatic parameter ranges in both sets of models were quite similar (Figure S1), with no range shifts and only a relatively moderate change in range size for Na_p_ and Kv_3.1_ densities (see Discussion).

### Models constrained by both BAC firing and perisomatic step current firing

Having successfully fitted the perisomatic step current firing and BAC firing features separately, we next attempted to do the same for their conjunction. Trying to simultaneously fit all 20 features for the two targets (Table 1) in a single MOO with high resources for the evolutionary algorithm (population size of 5000 and 2000 generations) did not yield satisfactory models for both targets, but rather generated models with large errors in one or several key features. This was to be expected with the large number of objectives, which requires a much larger population for convergence [46] (see Discussion). We therefore conducted the optimization process in two stages. The first stage was the fitting of BAC firing target, which resulted in the set of acceptable models shown in Figure 3 (red lines). We then tested this set of models on the features of perisomatic step current firing. Some models performed poorly, as demonstrated in Figure 1, whereas others performed somewhat better, although not as satisfactorily as the acceptable model for perisomatic step current firing shown in Figure 2. We thus selected the one acceptable model for BAC firing that performed best on the other target behavior as well, and used its specific dendritic parameter values in a new MOO on the perisomatic parameters alone, now constraining with both target behaviors. The fixation of dendritic parameters was based on the assumption that acceptable apical conductance densities do not influence the perisomatic step current firing significantly, as suggested in Figure S1. Indeed, this two-step method resulted in a successful fit, yielding acceptable models that faithfully reproduced the features of both target behaviors.

**Figure 4:**
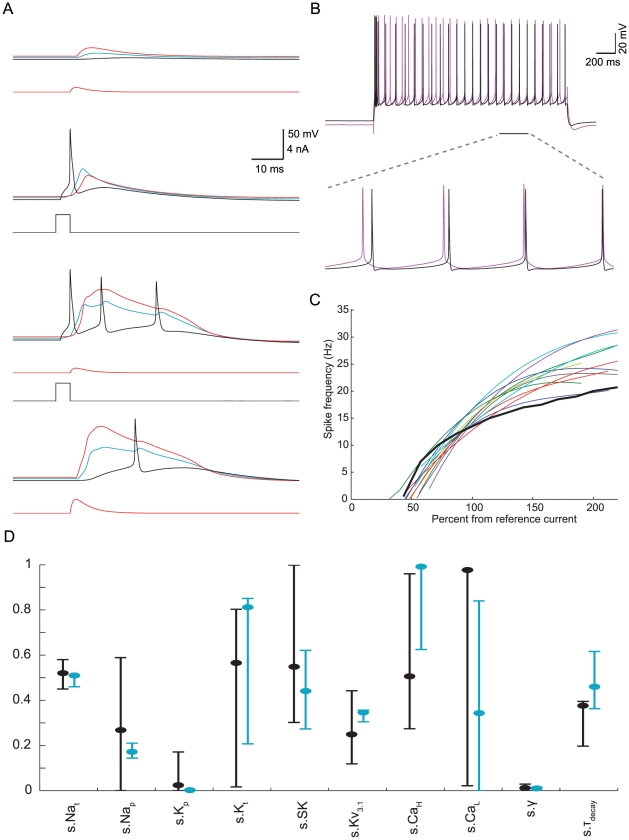
Example of an acceptable model for both BAC firing and perisomatic step current firing. **A**. The model faithfully reproduced BAC firing (compare to Figure 1B–E). **B.** Firing response of the same model as in (A) to perisomatic step current injection (black–model, magenta–experiment). **C.** The model's f–I curve (black) and experimental f–I curves (colors). All measures and definitions are as described in Figures 1 and 2. **D.** Distribution of the normalized parameter values in models targeted at only the perisomatic step current firing (black, n = 52), and in models that also fit the BAC firing target (blue, n = 4). The black and blue circles refer to the parameter values of specific models shown in Figure 2 and this figure, respectively. Ranges of some parameters in both cases overlapped, whereas the range of the Ca^2+^ τ_decay_ was markedly different in the two optimizations. Real values for the different ion conductances can be derived by referring to the upper limits given in Table 2.

An example of such a model is shown in Figure 4. This model was selected for having feature values closest to the experimental mean. The BAC firing that it produced (Figure 4A) was similar to that of the models exemplified in Figure 1, and the values of all BAC firing features were within 1–2 SD from the experimental mean, except that the average AP ISI was slightly longer (15 ms, ∼4 SD from the experimental mean). However, since we observed such a value in the experimental recordings data, we considered it acceptable. The model faithfully reproduced all the perisomatic step current firing features (Figure 4B) to within 1–2 SD from the experimental mean, except that its initial burst response was slightly stronger (comprising 3–4 spikes instead of 2–3). The model's f–I curve was within the experimental range (Figure 4C). Models in the set had feature values ranging within 2–3 SD from the experimental mean, thus exhibiting the experimental variability.

The f–I curve of the resulting models did, however, saturate at lower frequencies than average, so that the models captured the f–I curve of cells from the margins of our experimental set (Figure 4C). To explore the reason for this, we compared the somatic parameter ranges in the set of models for both BAC firing and perisomatic step current firing to the set of models fitting only perisomatic step current firing (Figure 4D). Apart from several decreases in range size (Na_p_, Na_t_, K_p_, and Kv_3.1_ densities), which may be attributed to the small data set of dually-constrained models, we noticed a marked increase in intracellular Ca^2+^ dynamics τ_decay_ (363–616 vs. 197–395 ms) in models that were capable of producing acceptable BAC firing, possibly due to the constraint of three APs in the perisomatic burst response during BAC firing (see Methods). τ_decay_ seems likely to underlie the stronger saturation of f–I curve since it is active on long time scales. We tested this hypothesis by lowering the τ_decay_ value in the model (460 ms) to be within the range of models for perisomatic step current firing alone (300 ms). We found that the f–I curve of the modified model indeed shifted to lie on the average (Figure S2, top). However, as expected, this modified model produced only two perisomatic APs in BAC firing (Figure S2, bottom). Hence, the constraint of three APs during BAC firing is likely to have clashed with the f–I curve constraint. We suggest that a more complex Ca^2+^ dynamics mechanism might improve the fit (see Discussion).

The model shown in Figure 4 had a membrane time constant of 10 ms, and we measured the input resistance at the soma to be 41.9 MΩ. Both values are within the experimental range [4]. The parameter values of that model are given in Table 3, and parameter values of the three additional acceptable models that agree with both targets are given in Table S1. As an indication that our model densities are biologically plausible, we observed that the dendritic Na_t_ density (107 pS/µm^2^) and the measured peak somatic Na_t_ current (50pA/µm^2^) are in the same order of magnitude of experimental estimates [12], [32]. As a demonstration that a feasible I_h_ is present in the apical dendrites, we verified that the model exhibits experimental findings [47] regarding the effect of I_h_ in attenuation of voltage along the dendrites (Figure S3). The slope of the curve when I_h_ is blocked (Figure S3B) is slightly less steep than seen experimentally, either due to the difference in dendritic morphology or the difference between a complete blocking of I_h_ in simulation compared to the limited extent of pharmacological blockade experimentally.

**Table 3:** Parameter values for the dually-constrained model shown in Figure 4.

Parameter	Value	Parameter	Value
s. 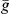 _ Nat_	20,400	ax. 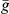 _leak_	0.325
s. 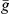 *_Nap_*	17.2	b. 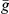 _leak_	0.234
s. 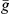 *_Kp_*	22.3	a. 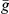 _leak_	0.295
s. 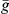 *_Kt_*	812	a. 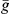 _Nat_	107
s. 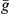 *_SK_*	441	a. 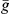 *_Kv3.1_*	1.31
s. 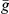 *_Kv3.1_*	6,930	a. 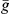 *_Ca_HVA_*	2.78
s. 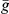 *_Ca_HVA_*	9.92	a. 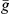 *_Ca_LVA_*	93.5
s. 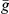 *_Ca_LVA_*	34.3	a. 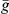 *_SK_*	6
s. γ	0.000501	a. 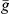 _m_	0.338
s.τ_decay_	460	a.τ_decay_	122
s.g_leak_	0.338	a. γ	0.000509

s–soma, a–apical, b–basal, ax–axon. Conductance is in pS/µm^2^, τ_decay_ in ms. Values for the apical Ca^2+^ channels are given for the “hot” zone. See Methods for fixed I_h_ values and passive parameters. The complete model is available in ModelDB [65] (accession number 139653).

### Testing the dually-constrained model on new stimuli and different morphologies

**Figure 5:**
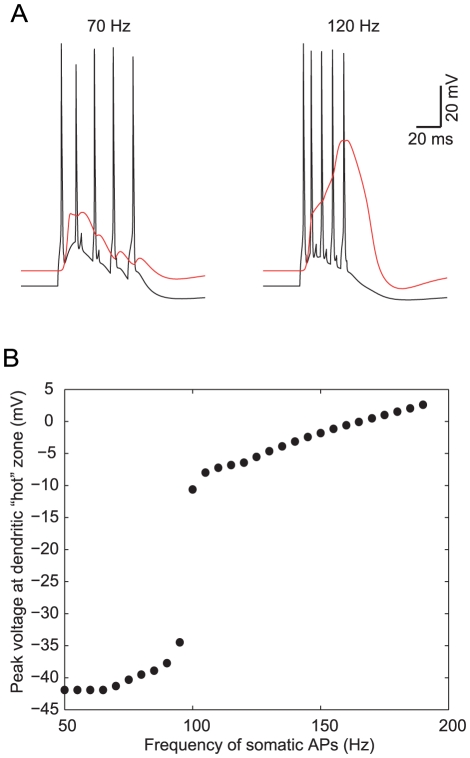
Ca^2+^ electrogenesis in distal apical dendrites in response to a critical frequency of somatic stimulation. **A.** The dually-constrained model neuron shown in Figure 4 was stimulated at the soma with a train of five brief suprathreshold depolarizing pulses, resulting in a train of somatic Na^+^ APs (black). Left, five somatic APs at 70 Hz did not elicit a Ca^2+^ spike in the dendrites whereas at 120 Hz (right) a regenerative Ca^2+^ spike was generated in the dendrites (red trace denotes dendritic voltage, measured at 830 µm from soma). **B.** Peak voltage response in the apical dendritic “hot” zone (830 µm from soma) as a function of the frequency of the somatic train of five APs. At a “critical frequency” around 100 Hz an “all or none” Ca^2+^ spike was generated at the dendrites.

We examined how well the dually-constrained model (shown in Figure 4) performs on a target behavior with which it was not explicitly constrained. Larkum et al. [23] have shown that when a train of APs is generated at the soma by a series of brief somatic pulses there is a critical frequency of somatic APs whereby the summated BAPs in the distal apical dendrites reach threshold for a regenerative dendritic Ca^2+^ spike. This critical frequency ranges in different L5b PCs from 50 Hz up to 200 Hz, with an average around 100 Hz. In Figure 5 we replicated this experiment by injecting a train of five brief suprathreshold current pulses to the soma of the dually-constrained model depicted in Figure 4. At frequencies below 100 Hz (Figure 5A, left) a dendritic Ca^2+^ spike was not elicited, whereas above 100 Hz a dendritic Ca^2+^ spike was generated (Figure 5A, right; and Figure 5B), in close agreement with the experimental results. This result strengthens our confidence in the model, as its good performance generalizes to novel experimental stimuli.

Next, we investigated the influence of the dendritic morphology on the results that we obtained for cell #1 (shown in Figure 1A). We selected two other L5b PC morphologies of the same age. One cell (cell #2) was generally similar to cell #1, while the other cell (cell #3) was more different than cell #1, in terms of input resistance (R_in_) and dendrite-to-soma conductance ratio [48] (ρ or “dendritic load”), or in the distance between the main apical bifurcation and the soma.

**Figure 6:**
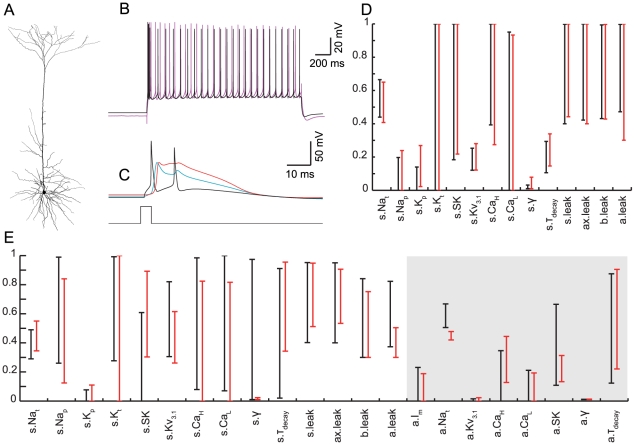
Model transfer to a similar morphology preserves perisomatic but not dendritic active properties. **A.** Reconstruction of a second L5b PC morphology of the same age as that of the cell in Figure 1A, with similar area and branching. **B.** Using the dually-constrained model shown in Figure 4 (and parameters shown in Table 3) with cell #2 provided an acceptable perisomatic step current firing (experimental trace–magenta; simulation–black). **C.** Brief suprathreshold somatic stimulation of the model with cell #2 generated BAC firing, in disagreement with the experimental results. **D.** Parameter ranges for acceptable models fitting perisomatic step current firing with cell #1 (black, n = 139) and cell #2 (red, n = 110) tightly overlapped. **E.** Parameter ranges for acceptable models fitting BAC firing with cell #1 (black, n = 899) and cell #2 (red, n = 948) generally overlapped, except for apical Na_t_ density. Real values for the different ion conductances can be derived by referring to the upper limits given in Table 2.

First, we used cell #2 (Figure 6A) with the same parameters (Table 3) as in the model shown in Figure 4. The main bifurcation in cell #2 was slightly more distal as compared to cell #1 (750 vs. 650 µm) although within the Ca^2+^ “hot” zone of cell #1; The R_in_ of cell #2 was smaller by 18% (34.4 vs. 41.9 MΩ) and its ρ was larger by 9% (16.1 vs. 14.7). The perisomatic firing response to step current (with amplitudes adjusted to the new R_in_) was acceptable and similar to that observed with the original morphology (Figure 6B). However, the dendritic tree was evidently more excitable, producing BAC firing upon a brief suprathreshold stimulation of the soma alone (Figure 6C). As is evident in that figure, the BAP amplitude at the distal apical dendrites was quite large in cell #2 as compared to cell #1 (Figure 1C), and was sufficient to trigger a Ca^2+^ spike (and consequently BAC firing). Based on previous studies [49]–[50] we measured the transfer impedance between the soma and the apical dendrite “hot” zone (700 µm away from the soma) in cells #1 and #2. For steady state, the transfer resistance was similar in the two cells (13.1 vs. 12.5 MΩ), however for a brief (5 ms) somatic pulse (corresponding to the duration of an AP) the transfer impedance was 1.4 times larger in cell #2 (1.714 vs. 1.2 MΩ), which explains why the somatic AP was less attenuated in cell #2 compared to cell #1.

We were interested in discovering ionic currents that compensate for the difference in transfer impedance. We therefore repeated the MOO fitting procedure of the BAC firing using the second morphology. We maintained the apical “hot” zone of Ca^2+^ channels at the same distance from the soma as for cell #1 (see Methods), since the main bifurcation differed only by 100 µm and was within the “hot” zone. We thus generated acceptable models for BAC firing in cell #2, and compared the ranges of their parameters to the set of acceptable models for cell #1 (Figure 6E). We found that acceptable models for cell #2 had a strictly lower apical dendritic Na_t_ density as compared to acceptable models for cell #1 (84–95.5 vs. 101–133.5 pS/µm^2^). This finding is in agreement with a previous study [50] that showed an inverse correlation between dendritic transfer impedance and the apical Na_t_ density required for the active BAP seen experimentally. Hence, when we used the parameters of the model shown in Figure 4 with cell #2, the apical Na_t_ was too high for that morphology to faithfully replicate BAC firing.

In addition, we checked if the successful transfer of somatic parameters between the two cells in terms of perisomatic step current firing was reflected in a similarity of somatic parameter ranges when cell #2 was fitted anew. We therefore repeated the MOO fitting procedure of the perisomatic step current firing using the second morphology. We used only leak and I_h_ conductance in the dendrites (as in generating the set of models for cell #1 shown in Figure S1, purple lines) to facilitate the comparison, and adjusted the step amplitudes according to R_in_. We thus generated acceptable models for perisomatic step current firing in cell #2. We found a tight overlap between their somatic parameter ranges and those of the corresponding models for cell #1 (Figure 6D), explaining the successful transfer from the cell #1 model to cell #2 in the case of perisomatic target behavior.

**Figure 7:**
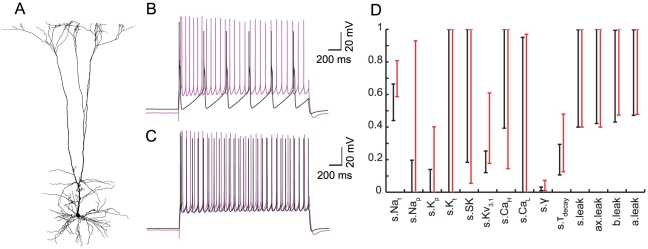
Model transfer between largely different morphologies is unsuccessful. **A.** Reconstruction of a third L5b PC morphology, cell #3, of the same age as cell #1 but with very proximal main bifurcation and larger dendritic tree area. **B.** Using cell #3 with the parameters of the acceptable model shown in Figure 4 did not yield a proper response even to somatic depolarization. **C.** Example of an acceptable model for perisomatic step current firing in cell #3. **D.** The parameter ranges of models for perisomatic step current firing using the morphology of cell #1 (black, n = 139) or cell #3 (red, n = 77) generally overlap, but not as tightly as for the second, more similar, morphology (Figure 6). Na_t_ and Kv_3.1_ ranges are shifted. Real values for the different ion conductances can be derived by referring to the upper limits given in Table 2.

The extreme effect of morphology on model performance is demonstrated by a third morphology, that of cell #3 (Figure 7A). Its main bifurcation was only 200 µm from the soma, its R_in_ was less than half that of cell #1 (18.8 vs. 41.9 MΩ) and its ρ was nearly 50% larger (20.2 vs. 14.7). When simulated using the parameters of the dually-constrained model for cell #1 shown in Figure 4, it fired incorrectly in response to somatic step current (Figure 7B). We observed similar poor transfer even for models with only leak and I_h_ conductance in the dendrites, which implied that the difference in the dendritic load underlies the poor transfer of parameters between the two modeled cells. While fitting anew for the perisomatic step current firing using cell #3 was successful (Figure 7C), acceptable models using cell #3 differed markedly from acceptable models that used cell #1, in that Na_t_ and Kv_3.1_ density ranges were shifted upwards (Figure 7D, 23,500–32,300 vs. 17,600–26,600, and 3,540–12,200 vs. 2,410–5,060 pS/µm^2^, respectively). Hence, these two conductances are likely to be important in adjusting for the dendritic load difference. A parameter range comparison between models for BAC firing was not possible, however, since the optimization of BAC firing with cell #3 did not converge well, regardless of whether assigning the apical Ca^2+^ channels high density using the distance rule (see Methods) or starting at the main bifurcation. This result suggests that there is a different channel distribution or BAC firing behavior for morphologies with such proximal main bifurcation. Overall, these results demonstrate some of the limitations of transferring models across L5b PC morphologies based on our current knowledge (see Discussion).

### Model prediction for BAC firing in Up vs. Down states

**Figure 8:**
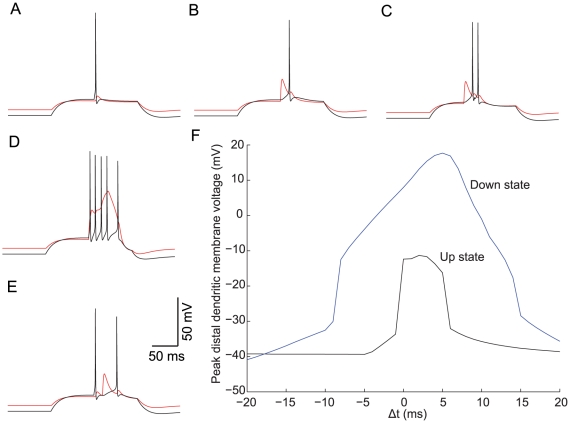
Enhanced gain and temporal sensitivity in BAC firing in Up state. Subthreshold DC depolarizing current of 0.42 nA was injected to the model shown in Figure 4, 200 µm from the soma, to emulate the Up state. Black–recording at the soma, red–recording at the apical dendrite, 700 µm from the soma. **A.** Model response to a brief, 5 ms 0.5 nA, step current pulse to the soma, resulting in a single AP. **B.** Model response when an EPSP-like current (0.5 nA) is injected in the main apical bifurcation, resulting in a single Na^+^ AP in the soma. **C–E.** Model response when the somatic and dendritic inputs coincide with time difference (Δt) of −20 msec (**C**), 0 msec (**D**) and +20 msec (**E**). Note that when the two inputs are applied with Δt of 0 (**D**), a Ca^2+^ spike is generated in the dendrite, along with a burst of five perisomatic Na^+^ APs. **F.** Peak dendritic voltage at the distal dendrite recording site as a function of Δt during an Up state (black) or Down state (when no DC current is applied, blue). Note the narrower time window for Ca^2+^ spike generation in an Up state.

We used our model to test the expected effect of the Up state as seen *in vivo* [51]–[52] on the BAC firing. Previous experimental studies [53] explored some of the properties of dendritic Ca^2+^ spikes and perisomatic Na^+^ spikes under noisy “high conductance” state. We emulated the Up state simply by applying a DC current of 0.42 nA for 200 ms at the proximal apical dendrite, 200 µm from the soma. Under this condition, a brief square pulse (5 ms, 0.5 nA) at the soma resulted in a perisomatic Na^+^ AP (Figure 8A). An EPSP-like current, similar to that used in our BAC firing simulations (0.5 nA), injected at the distal apical dendrite 700 µm from the soma (Figure 8B) resulted in a perisomatic Na^+^ AP, due to the proximity of the membrane potential to firing threshold in the Up state. When the EPSP input preceded or followed the somatic stimulus by 20 ms (Figure 8C and Figure 8E, respectively) the model cell fired two Na^+^ APs, with no spikes at the dendrite. However, when the two inputs were applied simultaneously, the cell BAC fired, with a burst of four additional Na^+^ APs as well as Ca^2+^ spike at the dendrite (Figure 8D). We then determined the temporal window conducive to the generation of Ca^2+^ spike, by plotting the peak distal dendritic membrane potential as a function of the stimulus time difference (Δt) in the Up state and the Down state (without the DC current, Figure 8F). The temporal window for the generation of the BAC firing was much narrower in the Up state (Δt = 0–5 ms, Figure 8F black trace) compared to the Down state (Δt = −10–15 ms, Figure 8F blue trace). Ca^2+^ spike peak was smaller in the Up state, in agreement with previous experimental studies [53]. Interesting also is the larger gain in the Up state (a larger number of additional BAC firing-related spikes) in the Up state compared to the Down state (see Figure 4). Therefore, our simulations predict an increase in temporal sensitivity for BAP and EPSP coincidence, as well as an increase in perisomatic AP gain during the Up state (see Discussion).

## Discussion

To the best of our knowledge, this is the first modeling study for any neuron type that utilizes an automated feature-based parameter search to faithfully replicate both dendritic Ca^2+^ and perisomatic Na^+^ electrogenesis and the interaction between these two spiking regions. In this study we modeled mature L5b PCs, focusing on the firing of Na^+^ APs at the soma in response to a prolonged step current, the generation of a Ca^2+^ spike at the distal apical dendrites, as well as the interaction between the two spiking zones via the BAC firing. To characterize these target behaviors, we used a total of 20 experimentally-based features and their experimental variability (Table 1). As a result, for a given modeled L5b PC morphology this study provides a set of acceptable models (and consequently a range of model parameters) that faithfully replicate the target experimental results, as well as exhibiting the experimental variability. Importantly, experimental studies also show that numerous combinations of ion channel densities can result in similar firing behavior [54]. Our sets of models can be used in further analyses that examine the interplay between channel density combinations. Dendritic I_h_ distribution in the models based on previous studies ensured replication of I_h_-related subthreshold input integration properties (e.g., Figure S3). The faithful performance of the models we present also generalized to stimuli with which they were not constrained (Figure 5), and the models perisomatic and dendritic maximal conductances are in the same order of magnitude as experimental estimates. Previous modeling studies of L5b PCs replicated only some aspects of the cell's behavior (e.g., BAC firing but not perisomatic step current firing [31], or *vice versa* [33]). Thus, our work provides a set of models faithfully replicating a range of important active properties of a key neuron in the mammalian neocortex, which can serve as a basic building block for *in silico* models of large-scale cortical networks.

In order to pinpoint key mechanisms that underlie the multi-regional firing properties of L5b PCs, we compared the range of parameters for models that optimized either of the two target behaviors. We sought parameters that differed between the two targets either in range size or in range values. Such differences provided a first order, readily-observable indication of the change in the role of a given ion channel and therefore hinted at its relevance to the target behavior. Also of interest were cases where the range of a parameter values included zero, indicating that this ion channel may be replaced by a combination of other ion channels for achieving the target behavior. Such comparisons provided a clear delineation of the range of apical Na_t_ and Kv_3.1_ density values required for a proper active back-propagation of APs, and also highlighted the K^+^ and Ca^2+^ related mechanisms affecting dendritic capability to generate local Ca^2+^ spikes (Figure 3). In particular, I_m_ and Kv_3.1_ may act directly to counter the regenerative effect of Ca^2+^ and Na^+^ currents, whereas Ca^2+^ dynamics γ may act by increasing the free Ca^2+^ concentration for a given Ca^2+^ current, thus triggering a larger SK current which dampens the local Ca^2+^ spike.

Through similar analysis we differentiated between somatic mechanisms that are primarily involved in shaping the AP, and somatic mechanisms that are more involved in features of the AP train. Ranges of values for parameters of somatic mechanisms with long time constants, such as SK, Ca_HVA_ and Na_p_ currents, and Ca^2+^ dynamics γ and τ_decay_, differed between the two sets of models. We verified that these mechanisms did indeed play a role in features of somatic spike trains such as adaptation. Such features were not sufficiently constrained by the BAC firing, which lasts only tens of milliseconds. By contrast, somatic parameter ranges that were similar in value were therefore likely to contribute to features of the perisomatic spike shape, which were constrained in both of our target behaviors.

Additionally, we highlighted the role of somatic Na_t_ density in compensating for changes in the dendritic load on perisomatic excitability in different L5b PC morphologies (Figure 7), as well as the role of dendritic Na_t_ density in compensating for changes in the transfer impedance between soma and apical dendrite in different L5b PC morphologies (Figure 6). We also found that active dendritic mechanisms do not seem to play a significant role in perisomatic step current firing (Figures 3, S1), except perhaps in contributing to the steady excitability involving the balance between persistent conductances such as dendritic Kv_3.1_, and somatic Na_p_ and Kv_3.1_ (Figure S1).

The range of values for perisomatic Na_T_ density in our models is somewhat higher than in several other estimates [32], [55]. In addition to the limitations of the experimental methods used for estimating ion channel density, which may underestimate the true density (as indicated by [32]), several reasons can account for the higher Na_T_ density used in our models. Some experimental estimates were based on younger preparations [55], where the cells (and thus the “dendritic load”) are considerably smaller than in mature cells [56]. Thus, models for younger cells require smaller density of perisomatic Na_T_ for generating “healthy” somatic Na^+^ spikes (as also suggested by Figure 7). Other estimates are based on model-fitting of only a single-spike in response to a brief current pulse [32]. Interestingly, we could also fit a single spike response using lower Na_T_ density (∼4,000 pS/µm^2^, not shown). However, a higher density was required to fit prolonged train of spikes. This is mostly due to the inactivation of Na^+^ channels during the plateau depolarization seen during firing response to prolonged current step. Our perisomatic Na_T_ density values agree with previous modeling studies of current step firing [30], [33]–[34]. Another critical factor that affects the estimates of Na_T_ channels density is the kinetics of the activation/inactivation of the channels. The estimates for kinetics parameters such as V_½_ values differ considerably in different studies [32], [55], [57], by 5–15 mV on average. A shift in V_½_ to more hyperpolarized values compensates for reduced channel density [29]. We therefore verified that the peak Na^+^ current measured at the soma in our models agrees with experimental estimates [32]. We also note that in the axonal model (Figure S2), the ratio of axonal to somatic Na_T_ densities agrees with recent experimental estimates [58].

In agreement with previous studies, we found that constraining one spiking zone did not guarantee the constraining of the other spiking zone [45]. In addition, the dissociation between the perisomatic AP features and the dendritic conductance mechanisms that underlie dendritic BAP is in agreement with previous experimental studies that blocked dendritic Na^+^ channels and showed no significant change in features of perisomatic APs in L5b PCs [12].

Previous modeling studies [47] suggest a non-uniform specific membrane resistance (R_m_) in the apical dendrites. In our study we used a uniform distribution. However, we verified that our models retain the faithful replication of the features in both soma and dendrites also for the case when R_m_ is spatially non-uniform. Specifically, we simulated the model shown in Figure 4, using the sigmoid function for R_m_, with R_m_(soma) = 34,963 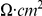, R_m_(end) = 5357 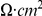, d_half_ = 406 µm, steep = 50 µm, with a factor of 1.16 to maintain the overall spatial integral of the leak conductance density as in our original model. In this case, in order to retain the resting membrane potential in the dendrites and the soma, we found it necessary to adjust E_leak_ across the apical dendrites, with E_leak_(soma) = −90 mV, E_leak_(end) = −80 mV, with similar d_half_ and slope as of R_m_.

In this study we did not attempt to fit for local dendritic Na^+^ spikes [5], [10], primarily to lessen the load on the optimization algorithm since their occurrence and function in the context of network activity in the intact brain seems to be minor [59] relative to Ca^2+^ spikes. Nor did we fit for active BAP in the basal dendrites since it does not differ substantially from passive propagation [10]. We also did not fit for dendritic NMDA spikes [8]–[9], [11] since, although they are an important signal, there is currently insufficient data with regard to the distribution and density of NMDA receptors. Once sufficient experimental data is available, the models presented hereby could serve as a scaffold on which NMDA spikes can be fitted for, using a similar automated optimization framework, since NMDA spikes do not depend significantly on voltage gated channels [9].

In the present study, the optimization parameters were the densities of the different ion channels. Our evolutionary algorithm framework can be used for exploring the effect of the kinetics of the different ion channels [60] as well as of different spatial distribution of ion channels in the soma-dendritic surface. Similarly, the powerful search algorithm can be used to refine our selected set of conductance mechanisms, e.g. when results imply that a certain mechanism is not necessary for fitting any of the targets, or that different mechanisms are required to improve the fit. For example, future optimization studies will benefit from a better model of intracellular Ca^2+^ dynamics, one that would account for a saturating buffer and a saturating pump. To our knowledge, a well-constrained model of such complexity does not yet exist. One major shortcoming of the simplistic and widely used model for Ca^2+^ dynamics that we employed in this study is that it acts to reduce intracellular Ca^2+^ concentration to a similar degree regardless of the concentration level. It therefore either acts strongly or weakly during both intense and weak stimuli.

Our study demonstrates the limitations in transferring an existing model to different morphologies. Even within the same general class (in our case, L5b PCs), different dendritic morphologies can have significant differences in the degree of electrical coupling between the two active zones (Figure 6, and see also [50], [61]) as well as differences in the dendrite-to-soma conductance ratio. Interestingly, when the dendrite-to-soma conductance ratio differs significantly, parameters that enable the fitting of perisomatic firing features in one cell fail to fit this target behavior in the other cell (Figure 7). Further parameter range comparison and more systematic investigations are needed to elucidate morphological effects on transferring models across morphologies. This will help in defining general rules for constructing generic models for a particular neuron type that are invariant to their morphological differences.

Previous *in vitro* studies [53] indicated invariance of the time window between BAP and dendritic EPSP conducive to BAC firing when comparing silent slice conditions (which can be considered a Down state) to when the cell is bombarded by intense dendritic input. Their suggested time window is ∼30 ms, in agreement with our simulation results for the Down state (∼25 ms). However, our model predicts a narrower time window (5 ms) during an Up state, a condition observed *in vivo* [51]–[52]. In our model, threshold for Ca^2+^ spike generation remained fixed in both our Up and Down states; therefore the reason for narrowing of the time window for BAC firing in the Up state is the reduction in the peak BAP recorded at the distal dendrite (reduction of ∼15 mV), due to inactivation of dendritic Na^+^ channels as a result of the DC depolarization. Our results thus complement the previous findings and suggest an enhanced sensitivity for input coincidence associated with increase in the BAC-firing related input-output gain–a prediction, which we hope, will be examined experimentally soon.

With the current computational resources in most neurobiology departments, multi-objective evolutionary algorithms of the kind we have used [62] are limited to fitting only a few objectives [46]. Optimization convergence seems to be further compromised when the objectives involve several highly nonlinear active zones that interact strongly with each other. For this reason, our method of fitting in stages (first the dendritic spiking zone, and then both spiking zones, Figure 4) can be useful for further optimization studies. It will be worthwhile to examine other MOO algorithms that may be better suited for a large number of objectives [63]–[64].

The sets of parameter and feature error values for all (∼2,000) models reported in this work are available in ModelDB [65], along with the relevant NEURON code (accession number 139653).

## Methods

### Optimization algorithm

We extended the algorithm described previously [36]. Briefly, statistics of electrophysiological features such as spike frequency, spike width, and adaptation index were grouped into multiple objectives and fitted to a detailed conductance-based model of a reconstructed L5b PC by an elitist non-dominated sorting evolutionary algorithm [62]. The free parameters in the optimization were primarily the density of a set of nine predefined ion channels (see below and Figures S4, S5) located in the soma and in the dendrites (Table 2). We divided the detailed reconstructed morphologies into compartments, each at most 20 µm long, resulting in an average of about 200 compartments per model cell. The algorithmic optimization and all simulations were conducted in NEURON [66]. For the evolutionary algorithm we used a population size of 1000 and 500 generations, running either on a grid of 60 Sun×4100 AMD 64 bit Opteron dual core (240 cores in total), running Linux 2.6, or on 1024 cores of an IBM BlueGene/P supercomputer hosted by CADMOS and accessible to the Blue Brain Project [67]. Runtime ranged from 2 to 5 days.

### Modeling

#### Passive properties

We set the membrane capacitance (C_m_) to 1 µF/cm^2^ for the soma and axon, and 2 µF/cm^2^ for the basal and apical dendrites to correct for dendritic spine area [68]–[69]. Specific membrane (leak) conductance (inverse of the specific membrane resistance, R_m_) was kept as a free parameter with limits doubled for the dendrites, corresponding to the capacitance modification. For the same reason, all dendritic conductance densities were doubled, although reported here in actual (not doubled) value. We set the axial resistance (R_a_) to be 100 Ω•cm for all compartments [47]. Leak reversal potential was set to −90 mV [13].

#### Perisomatic spike initiation zone

Although it is known that the action potential initiation zone in L5b PCs is at the axon [70], for simplicity we chose to reduce the multi-compartmental axon spiking zone into a single compartment zone located at the soma, to which we henceforth refer as the perisomatic spiking zone. In the reconstructed morphologies we kept only the initial axon segment, deleting the rest of the axon. However, we have also provided (see Text S1) a model with AP initiation in the axon rather than in the soma, which replicates faithfully the features of current step firing and of BAC firing, except that it is less successful in replicating the features related to the BAP (see Figure S6, Table S2).

#### Conductance mechanisms

We included ten key active ionic currents known to play a role in L5 PCs or generally in neocortical neurons [39], with kinetics taken strictly from the experimental literature. Kinetics of ion conductances that were characterized in room temperature (21°C) were adjusted to the simulation temperature of 34°C using Q_10_ of 2.3, and those taken from experiments where the junction potential was not corrected for were shifted by −10 mV. The reversal potentials for Na^+^ and K^+^ were E_Na_ = 50 mV and E_K_ = −85 mV, respectively, and a −45 mV reversal potential was used for the I_h_ current [13].

Ion currents were modeled using Hodgkin-Huxley formalism, so that for each ion current: 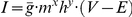



Where 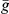 is the maximal conductance (or density); *x* and *y* are the number of gate activation and inactivation variables, respectively; *E* is the reversal potential of the ion involved; and *V* is the membrane potential.

The kinetics of the conductance mechanisms used in this study is detailed below (see also Figures S4, S5). Time constants are given in milliseconds (ms), voltage in millivolts (mV), and ion concentration in millimolar (mM). F is Faraday's constant; d is the depth of sub-membrane shell for concentration calculations in µm; γ is the inverse of the Ca^2+^ buffer's binding ratio; and τ_decay_ is the time constant of Ca^2+^ diffusion. 1e-4 mM refers to the steady state intracellular free Ca^2+^ concentration. The activation time constant of SK is estimated to be instantaneous (1 ms), since we could find no definite characterization of it in the literature due to the difficulty in measuring it experimentally.

*Fast inactivating Na^+^ current, I_Nat_* [57]:
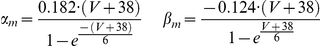


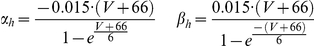


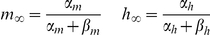


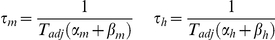


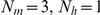



*Persistent Na^+^ current, I_Nap_* [71]:
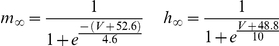


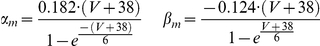


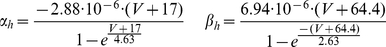


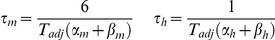


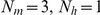



*Non-specific cation current, I_h_* [13]:
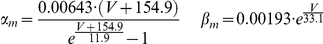


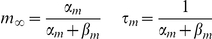


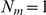



*Muscarinic K^+^ current, I_m_* [72]:
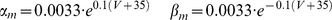


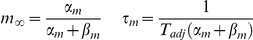


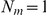



*Slow inactivating K^+^ current, I_Kp_* [73]: 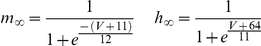


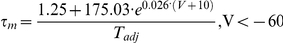


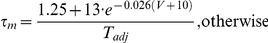


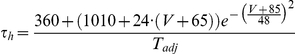


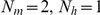



*Fast inactivating K^+^ current, I_Kt_* [73]:
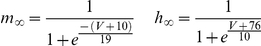


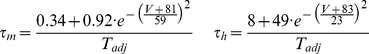


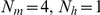



*Fast, non inactivating K^+^ current, I_Kv3.1_* [74]:
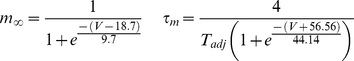


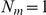



*Intracellular [Ca^2+^] dynamics* [35], [75]:
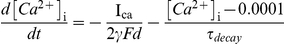



*High voltage activated Ca^2+^ current, I_Ca_HVA_* [76]:
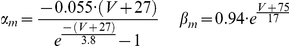


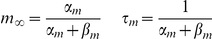


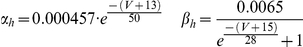


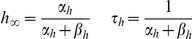


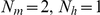



*Low voltage activated Ca^2+^ current, I_Ca_LVA_* [77]–[78] : 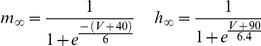


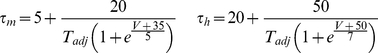


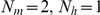



*Small-conductance, Ca^2+^ activated K^+^ current, I_SK_* [79]:
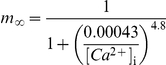


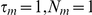



*Temperature adjustment factor:*

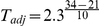



The optimization algorithm aimed at searching the densities for the ion channels (except for I_h_ which we fixed, see below) and the parameters of the Ca^2+^ buffer mechanism that best fit the target experimental features (see also [36]). The list of the free parameters and their limits used by the search algorithm is given in Table 2. The lower limits for density were 0, and upper limits were as high as biologically plausible.

#### Dendritic channel distribution

All dendritic channels except for I_h_, Ca_LVA_ and Ca_HVA_ were uniformly distributed. I_h_ channels were distributed on the apical dendrites using an exponential density function suggested in [13]: 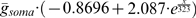, where *x* is the distance from the soma in µm, with 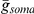 = 1 pS/µm^2^. The density of I_h_ on the basal dendrites was set to be uniform as suggested in [10].

Previous experimental studies [7] indicate that the low threshold zone for Ca^2+^ spikes in the apical dendrites might be located somewhere between 600 and 1000 µm from the soma, roughly from the main bifurcation to the end of the primary tuft. The main bifurcation in the reconstructed “typical” L5b PC (see below) that we used (Figure 1A) was 650 µm away from the soma and the primary tuft ended around 950 µm away from the soma. Ca_LVA_ and Ca_HVA_ were therefore distributed on the apical tree using a step function, with increased conductance between 685 and 885 µm from the soma. This high density (“hot”) Ca^2+^ zone had 100 and 10 times higher density of Ca_LVA_ and Ca_HVA_ than anywhere else on the apical tree.

### Experimental data

We calculated statistics for the features of perisomatic step current firing directly from experimental voltage traces of the firing response to step currents in adult, P36 Wistar rats measured *in vitro* (see [4] for methods). Briefly, cells were injected with depolarizing current of variable amplitudes, each 2 seconds in duration, and recorded in whole-cell configuration at 33–35°C. We used data from 11 L5b PCs, each with 10–15 different current step amplitudes that were repeated twice. These cells were also stained with biocytin and some were 3D reconstructed under light microscopy in Neurolucida (Microbrightfield). All morphologies have been checked for z-axis noise, improper diameters, and corrected for tissue shrinkage. We chose a cell with a typical response (see below) and morphology to be used as our reference cell for fitting and simulations (Figure 1A). We used two other cell reconstructions for investigating how the models generalized across different morphologies (Figures 6A, 7A). According to experimental studies [56], pyramidal cells at age P36 are mostly mature in terms of electrical properties, morphology and the ability to generate dendritic Ca^2+^ spikes. For the BAC firing features, we used statistics reported in [6] as well as our own calculations from voltage traces of five cells that were kindly provided by M. Larkum. BAP attenuation features were characterized using statistics derived from the literature [44].

### Extracting spiking features from experimental data

We used a set of key features of target firing behavior at the soma and dendrites (Table 1). These served as the objectives to be fitted by the evolutionary algorithm. The error in each feature was measured in terms of SD from the experimental mean for that feature.

#### Features of perisomatic step current firing

For the perisomatic firing response to step current, some features of interest were defined as in [36]. We added the following features:

- *Inter-spike interval coefficient of variance (ISI-CV)*: defined as 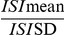

- *Initial Burst ISI:* the length (in ms) of the ISI between the first two spikes, which in these cells is typically much smaller than that of the rest of the spikes, and is considered as a burst.
- *Mean fast and slow after-hyperpolarization (AHP) depth:* the minimum voltage between two spikes in the train. Due to the occurrence of two types of AHP, fast and slow, which correspond approximately to before and after the first 5 ms of the ISI, we defined two separate features for the AHP.
- *Mean slow AHP time:* the time (relative to the ISI duration) of the minimum of the slow AHP. This feature complements the previous feature in characterizing the shape of the voltage trace between spikes.
- *f-I curve:* we normalized the f-I curves of the different experimental cells we used in this study (Figure 2B), which differ in their R_in_ value, by defining a ‘reference frequency’ of 15 Hz (the middle, more linear, part of the f–I curve) with the corresponding reference step current that produced this frequency. We then selected two additional normalized current amplitudes for which we had sufficient data (which were 78% and 190% of the reference current) to quantify the variability of spike rate in low, medium and high firing frequencies. We also used the corresponding voltage traces for the three current amplitudes in quantifying the statistics of the other features mentioned above.

The current amplitudes used in the optimization algorithm were the averages across cells. The primary morphology we used (Figure 1A) was that of a cell that fired at the reference frequency (15 Hz) in response to a current of amplitude that was near the average across all eleven cells. This cell also had feature values that were close to the average across cells, and was therefore considered “typical”.

#### Features of BAC firing

We constrained the distal apical spiking zone to exhibit experimentally observed BAC firing [6] that included: (a) a proper somatic AP when the soma is injected with brief suprathreshold step current; (b) an attenuated BAP; and (c) the generation of a dendritic Ca^2+^ spike accompanied by additional somatic Na^+^ APs when the somatic injection coincided with distal dendritic EPSP-like current stimulation.

The ten features characterizing BAC firing (Table 1) included dendritic features as well as somatic spike shape features similar to those described for the perisomatic step current firing.

- *Somatic AP spike count, during soma current injection only:* ensuring exactly one spike for a brief suprathreshold pulse.
- *BAP amplitude, at 620 and 800* µ*m from the soma:* was constrained according to experimental findings [44]. These two points were chosen since they delineate the “hot” zone, from the distal apical trunk to the furthest distance for which experimental data was available.
- *Somatic AP count, during soma and dendrite current injection:* strictly ensuring a burst of three somatic APs, since that case was more frequent than the case of two somatic APs in the experimental data we used, and also involved more clearly defined Ca^2+^ spikes.
- *Mean somatic ISI:* the average ISI of the somatic APs during BAC firing.
- *Dendritic Ca^2+^ spike peak:* the maximum dendritic voltage during the Ca^2+^ spike in the distal trunk.
- *Dendritic Ca^2+^ spike width:* was computed at the base of the spike (−55 mV) since it was a consistent feature of all Ca^2+^ spikes (unlike the width at half-height).

The values for the BAC firing features were derived from five somatic and dendritic voltage traces that were previously reported [6], or directly from values reported in that article.

#### Objectives number

There is a critical advantage in reducing the number of objectives that our evolutionary algorithm has to optimize both in terms of required resources and convergence [46]. We combined some of the features into single objectives (see Text S2), resulting in eight objectives for fitting the perisomatic step current firing and five objectives for fitting BAC firing.

#### Acceptable models and parameter ranges

From the entire population in each optimization, we selected as acceptable models ones that had all feature values within 2–3 experimental standard deviations from the corresponding experimental mean. Our resultant sets of models comprised tens to hundreds of models from at least two (and often three or four) runs using different randomizations to avoid single run effects on correlations between the model parameters. We used the sets of models to delineate “acceptable” parameter value ranges for each target. Adding models from further runs did not change the parameter ranges significantly, therefore we considered the ranges a good approximation of the complete ranges. For ease of visualization, we presented normalized parameter values so that 1 corresponds to the upper limit given to the algorithm (see Table 2 for values).

## Supporting Information

Figure S1 **Parameter ranges in acceptable models for step current firing are independent of active dendritic mechanisms.** Distribution of normalized parameter values in models of perisomatic step current firing with active dendritic mechanisms (black, n = 52) or with only I_h_ and leak conductance in dendrites (purple, n = 139). Real values for the different ion conductances can be derived by referring to the upper limits given in Table 2.
(EPS)

Figure S2 **Trade-off between fitting f-I curve and BAC firing.** When we lowered the Ca^2+^ dynamics τ_decay_ in the model shown in Figure 4, its f-I curve shifted closer to the average (top), but the modified model produced two APs instead of three during BAC firing (bottom). See Figures 1 and 2 for measures and definitions.
(EPS)

Figure S3 **Effect of I_h_ on voltage attenuation in the apical dendrites.** **A.** The response of the model shown in Figure 4 to a −50 pA, 200 ms square current injected at the soma in control conditions (top) and when I_h_ is blocked (bottom). Note the sag current typical to I_h_. (Black – soma, Red – main apical dendrite bifurcation). **B.** Steady state voltage attenuation along the apical dendrites in control condition (black) and when I_h_ is blocked (red).
(EPS)

Figure S4 **Channel kinetics curves, activation/inactivation.** Activation/inactivation curves for the ionic channels used in this study. See Methods for kinetics equations. **A.** Fast inactivating Na^+^ current, *I_Nat_*. **B.** Persistent Na^+^ current, *I_Nap_*. **C.** Non-specific cation current, *I_h_*. **D.** Small-conductance, Ca^2+^ activated K^+^ current, *I_SK_*. **E.** Slow inactivating K^+^ current, *I_Kp_*. **F.** Fast inactivating K^+^ current, *I_Kt_*. **G.** High voltage activated Ca^2+^ current, *I_Ca_HVA_*. **H.** Low voltage activated Ca^2+^ current, *I_Ca_LVA_*. **I.** Muscarinic K^+^ current, *I_m_*. **J.** Fast, non inactivating K^+^ current, *I_Kv3.1_*_._
(EPS)

Figure S5 **Channel kinetics curves, time constants.** Time constant curves for the ionic channels used in this study. See Methods for kinetics equations. **A.** Fast inactivating Na^+^ current, *I_Nat_*. **B.** Persistent Na^+^ current, *I_Nap_*. **C.** Non-specific cation current, *I_h_*. **D.** Slow inactivating K^+^ current, *I_Kp_*. **E.** Fast inactivating K^+^ current, *I_Kt_*. **F.** High voltage activated Ca^2+^ current, *I_Ca_HVA_*. **G.** Low voltage activated Ca^2+^ current, *I_Ca_LVA_*. **H.** Muscarinic K^+^ current, *I_m_*. **I.** Fast, non inactivating K^+^ current, *I_Kv3.1_*_._
(EPS)

Figure S6 **Dually-constrained model with AP initiation at the axon.** **A.** The model replicates faithfully all features of BAC firing except that its BAP attenuation is stronger and the BAP is too broad compared to the experimental results. **B.** The model replicates faithfully all features of perisomatic current step firing. All measures and definitions are as in Figures 1, 2 and 4. Parameters are provided in Table S2.
(EPS)

Table S1 **Parameter values of additional models for both BAC firing and perisomatic step current firing.**
(DOC)

Table S2 **Parameter values of model with AP initiation in the axon.**
(DOC)

Text S1 **Supporting results.**
(DOC)

Text S2 **Supporting methods.**
(DOC)
