# Supplementary material for: Models of Neocortical Layer 5b Pyramidal Cells Capturing a Wide Range of Dendritic and Perisomatic Active Properties
Source: PLoS Comput Biol. 2011 Jul 28;7(7):e1002107. doi: 10.1371/journal.pcbi.1002107 (PMC3145650; doi:10.1371/journal.pcbi.1002107)
Supplement: Table S1 — Parameter values of additional models for both BAC firing and perisomatic step current firing. (DOC) [file pcbi.1002107.s007.doc]

**Table S1. Parameter values of additional models for both BAC firing and perisomatic step current firing.**

| **Parameter** | **Model 1** | **Model 2** | **Model 3** |
| --- | --- | --- | --- |
| s._Nat_ | 20,100 | 18,400 | 20,400 |
| s.*_Nap_* | 21 | 14 | 21 |
| s.*_Kp_* | 1 | 16 | 22 |
| s.*_Kt_* | 801 | 207 | 851 |
| s.*_SK_* | 273 | 621 | 370 |
| s.*_Kv3.1_* | 7,120 | 6,090 | 6,930 |
| s.*Ca_HVA* | 8 | 6 | 10 |
| s.*Ca_LVA* | 84 | 0 | 34 |
| s.γ | 0.0005 | 0.0008 | 0.0005 |
| s.τ_decay_ | 616 | 363 | 494 |

Three additional models from the set that included the model shown in Figure 4. Conductance is in pS/µm^2^, τ_decay_ is in ms. s – soma. Values for the leak conductance and dendritic parameters are identical to those given in Table 3. See Methods for fixed values of I_h_ and passive parameters.
