# Supplementary material for: Models of Neocortical Layer 5b Pyramidal Cells Capturing a Wide Range of Dendritic and Perisomatic Active Properties
Source: PLoS Comput Biol. 2011 Jul 28;7(7):e1002107. doi: 10.1371/journal.pcbi.1002107 (PMC3145650; doi:10.1371/journal.pcbi.1002107)
Supplement: Table S2 — Parameter values of model with AP initiation in the axon. (DOC) [file pcbi.1002107.s008.doc]

**Table S2. Parameter values of model with AP initiation in the axon.**

| **Parameter** | **Value** | **Parameter** | **Value** |
| --- | --- | --- | --- |
| ax.$\bar{g}$ _Nat_ | 38,961 | s.$\bar{g}$_m_ | 0.08 |
| ax.$\bar{g}$*_Nap_* | 58.34 | a.$\bar{g}$_Nat_ | 107.45 |
| ax.$\bar{g}$*_Kp_* | 1,888.5 | a.$\bar{g}$*_Kv3.1_* | 9.04 |
| ax.$\bar{g}$*_Kt_* | 772.74 | a.$\bar{g}$*Ca_HVA* | 3.51 |
| ax.$\bar{g}$*_SK_* | 0.47 | a.$\bar{g}$*Ca_LVA* | 709.75 |
| ax.$\bar{g}$*_Kv3.1_* | 4,738 | a.$\bar{g}$*_SK_* | 0.01 |
| ax.$\bar{g}$*Ca_HVA* | 2.222 | a.$\bar{g}$_m_ | 4.95 |
| ax.$\bar{g}$*Ca_LVA* | 8.13 | a.τ_decay_ | 35.7 |
| ax.γ | 0.000525 | a.γ | 0.000637 |
| ax.τ_decay_ | 277.3 | ax.g_leak_ | 0.3 |
| ax.$\bar{g}$_m_ | 133.22 | s.g_leak_ | 0.3 |
| s.$\bar{g}$ _Nat_ | 9,989 | a.g_leak_ | 0.3 |
| s.$\bar{g}$*_Kv3.1_* | 3,380 | b.g_leak_ | 0.3 |
| s.$\bar{g}$*_SK_* | 996.5 | ax.g_h_ | 1 |
| s.$\bar{g}$*Ca_HVA* | 6.44 | s.g_h_ | 1 |
| s.$\bar{g}$*Ca_LVA* | 5.57 | a.g_h_ | 0.5 |
| s.γ | 0.000509 | b.g_h_ | 0.5 |
| s.τ_decay_ | 294.7 |  |  |

s – soma, a – apical, b – basal, ax – axon. Conductance is in pS/µm^2^, τ_decay_ is in ms. Values of apical Ca^2+^ channels are given for the high density distal zone.
