# Supplementary material for: Models of Neocortical Layer 5b Pyramidal Cells Capturing a Wide Range of Dendritic and Perisomatic Active Properties
Source: PLoS Comput Biol. 2011 Jul 28;7(7):e1002107. doi: 10.1371/journal.pcbi.1002107 (PMC3145650; doi:10.1371/journal.pcbi.1002107)
Supplement: Text S1 — Supporting results. (DOC) [file pcbi.1002107.s009.doc]

**Supporting Results**

Models with AP initiation at the axon

Using our automated optimization framework, we have produced additional models having the AP initiation at the axon. The axonal compartments encompassed the initial segment (60 µm), over which channels were distributed uniformly. Somatic Na_t_ channel kinetics was shifted by 6 mV, as suggested by experimental findings [57]. The models replicate faithfully all features of the step current firing and BAC firing, except that the BAP amplitude is too attenuated, and its half-width is much larger than the experimental average. For this reason we favoured the models with AP initiated at the soma. Nevertheless, we offer these additional models for the benefit the interested readers. An example model, which had feature values closest to the experimental means, is shown in Figure S6. Model parameters are given in Table S2.
