# Supplementary material for: Models of Neocortical Layer 5b Pyramidal Cells Capturing a Wide Range of Dendritic and Perisomatic Active Properties
Source: PLoS Comput Biol. 2011 Jul 28;7(7):e1002107. doi: 10.1371/journal.pcbi.1002107 (PMC3145650; doi:10.1371/journal.pcbi.1002107)
Supplement: Text S2 — Supporting methods. (DOC) [file pcbi.1002107.s010.doc]

**Supporting Methods**

**Objectives.** To reduce the number of objectives that our evolutionary algorithm had to optimize, we combined the three values of each perisomatic step current firing feature (corresponding to three different step amplitudes) into a single objective. In addition, the AI and ISI-CV features were combined into a single objective. Similarly, the slow and fast AHP depth features were combined into a single objective. Thus, eight objectives were used for fitting the perisomatic step current firing.

BAC firing features were combined into five objectives: (1) the somatic features (AP count, peak and width) during brief suprathreshold stimulation of the soma alone; (2) the BAP amplitude in two dendritic locations; (3) the Ca^2+^ spike shape (height and width) during somatic and dendritic stimulation; (4) the somatic features (AP peak, AHP depth and ISI) during somatic and dendritic stimulation; and (5) the AP count during somatic and dendritic stimulation.

In the optimization, the error value for each objective was defined to be the maximum of the individual feature errors in that objective, in order to guarantee an upper bound for the error of each of the individual features.
